# Comparative analysis of dietary vs. non-dietary approaches in obesity and disordered eating behaviors: a narrative review of the literature

**DOI:** 10.1007/s40519-024-01702-3

**Published:** 2024-11-26

**Authors:** Chiara Breda, Adriana Chiarelli, Giulia Quarantelli, Maria Vittoria Conti, Nagaia Madini, Hellas Cena

**Affiliations:** 1 Laboratory of Dietetics and Clinical Nutrition, Department of Public Health, Experimental and Forensic Medicine, University of Pavia, Via Bassi 21, 27100 Pavia, Italy; 2 Clinical Nutrition Unit, General Medicine, ICS MAUGERI IRCCS, Pavia, Italy

**Keywords:** Intuitive eating, Mindful eating, Non-dietary approach, Dietary-approach, Obesity, Disordered eating

## Abstract

**Purpose:**

This narrative review aims to conduct a comparative analysis of dietary and non-dietary approaches in the management of weight and disordered eating behaviors (DEBs) in adults with obesity.

**Methods:**

Studies were identified from Medline (PubMed), including only English-language manuscripts published from 1998 to 2024. To be included in the review the studies had to be RCTs that compared the effect of dietary and non-dietary approaches on weight loss and DEBs in adults with obesity not being treated with pharmacological treatments.

**Results:**

Seven randomized controlled trials (RCTs) reported in 8 manuscripts published between 1998 and 2024 met the inclusion criteria. The sample size ranged from a minimum of 16 subjects to a maximum of 219. All studies involved adult subjects, mainly women, with first-, second-, or third-degree obesity and most subjects had cognitive restriction and/or uncontrolled eating. Dietary approaches were characterized by moderate caloric restriction (CR) and restriction of fat intake. In contrast, non-dietary approaches focused on mindful eating (ME), intuitive eating (IE) and weight neutral (WN) approaches. Of the 7 RCTs included, 5 reported greater weight loss in the diet group compared to the non-diet group; however, only one of these sustained the result at follow-up. In contrast, 4 studies demonstrated greater improvements in DEBs in the non-diet group.

**Conclusion:**

CR is essential for weight loss in individuals with obesity, but long-term weight management also hinges on their relationship with food. The psychological improvements reported in non-dietary versus dietary approaches should not be overlooked and can be a starting point for the development of multidisciplinary interventions involving synergistic actions between diet, exercise, and practices to improve DEBs with the goal of reducing the obesity epidemic.

*Level of evidence* Level I, at least one properly designed randomized controlled trials; systematic reviews and meta-analyses; experimental studies.

## Introduction

Obesity is a multifaceted, chronic, and recurring condition characterized by excess accumulation of body fat, significantly heightening the risk for comorbidity [1]. Diagnosis is usually based on the assessment of body mass index (BMI), which if > 30 kg/m^2^ determines a status of obesity [2]. Despite extensive efforts to reduce its prevalence, overweight and obesity persist as ongoing challenges in global health [3, 4] and adiposity has been consistently shown to be an independent risk factor for many non-communicable diseases (NCDs) [5]. The economic burden of obesity, estimated at 2 trillion USD annually, underscores the urgent need for effective interventions [6].

Despite the availability of various dietary interventions [7], there is still no consensus on the most optimal dietary pattern for weight loss and long-term management.

In fact, traditionally, efforts to address obesity through diets focus on calorie restriction (CR) [5]. However, it is important to point out that, according to the goal conflict theory, the tension between craving for enjoyable foods and the pursuit of long-term weight-loss objectives, coupled with constant reminders to eat in today’s food-centric surroundings, paves the way for lapses in self-control. This combination of intrinsic and extrinsic factor ultimately results in disinhibited eating, feeling of guilt, and weight cycling syndrome [8–10]. In fact, weight regain in people with obesity is typically expected, with one-third to two-thirds of the lost weight being regained within the first year, and nearly all of it being regained within 5 years [11]. Thus energy-restricted diets may trigger negative repercussions, leading the patient to an increased risk of developing Disordered Eating Behaviors (DEBs) and eating disorders (EDs) [9, 10, 12]. EDs are diagnosed according to specific criteria defined in the Diagnostic Manual of Mental Disorders, Fifth Edition (DSM-5), whereas DEBs describe a range of irregular eating behaviors that may or may not warrant a diagnosis of a specific ED [13, 14]. DEBs, however, remain a significant medical problem, correlated with an increased risk of developing EDs, depression, alcohol and tobacco use, inadequate nutritional intake and quality, and significant long-term weight gain [15]. Not surprisingly, DEBs such as uncontrolled eating (an inability to inhibit eating once started), emotional eating (overeating in response to internal cues), and cognitive restriction (attempts at deliberate and persistent food restriction) are prevalent among obese individuals [16].

On the basis of what has been mentioned above, considering overweight/obesity and DEBs as existing along a spectrum rather than as separate and opposing conditions, has been suggested as a potentially more efficacious approach to their treatment and prevention [17]. In this scenario, interventions emphasizing present-moment awareness without judgment such as Mindful Eating (ME), and approaches fostering a positive relationship with food and body image including Intuitive Eating (IE) and weight-neutral (WN) programs, have shown promise in reducing DEBs across various studies [8]. In fact, Hackett et al. in 2022 [18] published a systematic review with meta-analysis of 14 intervention studies in which IE was observed to promote favorable change in the eating behavior of adult subjects with obesity and normal weight. Moreover, Yu et al. [19] published a systematic review which included 9 RCTs employing ME as a primary intervention for people both with obesity and normal weight with eating or body image problems, showing that this approach resulted in significant reductions in emotional eating, external eating, binge eating, and preoccupation with weight and shape.

Getting more specific about these approaches, ME roots from mindfulness-based approaches which have been proven to be effective in treating a broad range of clinical disorders and symptoms such as anxiety, depressive symptoms, stress, quality of life and psychological or emotional distress [20]. ME involves attentively focusing on food during consumption, cultivating awareness, and directing attention to the food experience. This approach is based on increasing mindfulness during eating occasions may enhance awareness of food consumption, potentially leading to the adoption of healthier dietary choices [21].

Different from the ME approach is IE, which involves consciously prioritizing physiological hunger and satiety cues over external stimuli when regulating food consumption [21]. External stimuli include emotions, food accessibility, visual or olfactory food cues, social contexts promoting eating behavior, portion sizes, and food packaging. IE requires training individuals to attune to bodily sensations (innate cues) to ascertain nutritional requirements [21]. Specifically, IE is based on ten principles: Reject the Diet Mentality, Honor Your Hunger, Make Peace with Food, Challenge the Food Police, Respect Your Fullness, Discover the Satisfaction Factor, Honor Your Feelings Without Using Food, Respect Your Body, Exercise-Feel the Difference, Honor Your Health [22].

Finally additional non-diet approaches besides ME and IE are the so-called WN approaches to health, which are based on mindfulness skills and emphasize IE, self-care, enjoyable exercise, and body-size acceptance. These approaches do not require reductions in BMI, thereby reducing the inherent stigmatization associated with recommending weight loss for individuals with a high BMI [23].

Since effectiveness and feasibility of non-dietary approaches (ME, IE, WN) on the management of obesity condition and DEBs, require further research, this narrative review aims to conduct a comparative analysis of dietary and non-dietary approaches in the management of weight and DEBs in adults with obesity.

## Materials and methods

This narrative review began in February 2024 with a nonsystematic search. The included studies had to be RCTs that compared the effect of dietary and non-dietary approaches on weight loss and DEBs in adult subjects with obesity not being treated with pharmacological treatments. Studies were identified from Medline (PubMed), including only English-language manuscripts published from 1998 to 2024.

The authors reported the following keywords: intuitive eating, mindful eating, bulimia nervosa, anorexia nervosa, binge eating disorder, BED, non-dietary approach, dietary-approach, purge, purging, obesity, disordered eating, eating disorder(s), calorie restriction.

The full texts of a total of 30 studies were screened, and 22 were excluded on the basis of the following criteria: (i) they were not RCTs; (ii) they did not compare a dietary approach with a non-dietary approach; (iii) It was not clear whether calorie or macronutrient restriction was implemented in the dietary approach.

## Results

In line with the inclusion and exclusion criteria of the study, 8 manuscripts were included in the review.

### Characteristics of the included studies

The selected studies were 7 RCTs reported in 8 articles [23–30] published between 1998 and 2024. The studies were conducted in the United States and Brazil. The sample size ranged from a minimum of 16 [24] subjects to a maximum of 219 [28]. All studies involved adult subjects, mainly women, with first-, second-, or third-degree obesity.

Regarding the approaches compared, all studies reviewed compared dietary and non-dietary approaches in two separate groups of population with obesity, only one study [28] compares these two approaches to a third (waitlist control group). All dietary approaches were characterized by a moderate CR with the exception of one study [28] that specified only a restriction of fat intake to 40 g/day; two studies reported a combined CR and fat restriction [25, 26]. Regarding the extent of the calorie deficit, two studies reported a caloric deficit of 500 kcal/day [24, 30], three studies did not specify the extent of CR [23, 25, 26], and one study used the formula 20 kcal/kg/day [27] to determine the caloric deficit. Finally, four studies combined dietary restriction (either caloric or fat restriction) with the LEARN program for weight control [23, 25, 26, 28], a program focused on modifying diet and lifestyle, with an emphasis on weight loss as the ultimate goal [23]. In contrast, non-dietary approaches focused on ME [30], IE [24, 27] and WN-programs [23, 25, 26, 28, 29].

With respect to the eating behavior of the subjects included in the studies, most included subjects/patients with cognitive restriction and/or uncontrolled eating [23, 25–30], no studies included patients with an eating disorder diagnosed according to DSM-5 criteria, and one study [24] did not specify the eating behaviors of the subjects/patients.

The interventions ranged from 1.5 [24] to 18 months [23], with the closest follow-up after the end of the intervention being after 1 month [25], and the most spaced-out follow-up being after 18 months [23].

Only one study [24] did not include a follow-up and greater weight loss was observed in the diet group compared to the IE group, though this effect was limited to the intervention period.

Of the 6 studies which reported follow-up, only 1 [25] reported significantly greater weight loss at follow-up in the dietary approach group compared with the non-dietary one. Three studies [23, 26, 28] found that statistical significance of weight loss was lost at follow-up in the group following the dietary-approach as weight lost during the intervention was regained. In two studies [27, 30], on the other hand, no difference in weight loss was found between the two approaches. Regarding eating behaviors, of the 6 studies that considered this parameter, 4 studies [23, 25, 26, 29, 30] reported a significant improvement at follow-up in susceptibility to hunger, disinhibition, uncontrolled eating, emotional eating, and DEBs in the groups following the non-dietary approaches HAES, HUGS and IE compared with the dietary-approaches. In contrast, the other two studies [27, 28] did not report statistically significant differences between the two approaches. Attrition was similar between the diet and non-diet approaches in three studies [23, 27, 28]. In contrast, two studies [25, 26] reported a higher dropout rate in the diet approach, whereas one study [30] indicated a higher dropout rate in the non-diet approach. Additionally, one study [24] did not report any data on dropout rates.

To help the reader in comparing their studies, the authors inserted Table 1 in which they specified the following aspects:

- country where the study was carried out;
- study design;
- age and sex of the enrolled subjects, and distribution of the sample between the two approaches;
- the average BMI of the enrolled subjects;
- type of dietary approach (caloric and/or nutrient restriction);
- type of non-dietary approach (IE/ME/WN-approach);
- DEBs evaluated in the study and questionnaires used;
- duration of the intervention;
- time spent from the end of the intervention to the follow-up assessment and attrition;
- the effect of dietary and non-dietary approach on weight loss and DEBs.

**Table 1:** Characteristics of the included studies

	Country	Study design	Age, sex and distribution of the sample	BMI of the sample	Dietary approach	Non-dietary approach	DEBs evaluated and questionnaire used	Duration	Follow-up and attrition	Results
Godrick et al. [28]	USA	RCT	219 adult women aged 40 ± 6.3 randomized to Diet Group (*n* = 79) Non-diet group = *n* = 78), and wait list control group (*n* = 62)	33.0 ± 3.4	Restriction of fat intake to 40 g/day + LEARN program for weight control	WN program	Risk to develop binge eating disorder (BES)	Both groups attended 24 weekly, 1-h group sessions for 6 months followed by 26 biweekly maintenance classes for 12 months	At the end of the maintenance classes. 14 participants in the DT group and 16 participants in the NDT group did not complete the interventions	The diet group lost significantly more weight than the non-diet group (*p* < 0.04) The diet and non-diet groups showed similar reductions in binge-eating scores after the 24 weekly 1-h group sessions (− 12.40 and − 10.29, respectively, *p* = 0.27) At the end of the maintenance classes, both the diet and non-diet groups had similar gains of more than 1 kg above baseline (*p* = 0.85) and showed similar net decreases from baseline in BES scores (− 13.57 and − 12.68, respectively, *p* − 0.66)
Bacon et al. [25]	USA	RCT	78 adult Caucasian women aged 39.3 ± 4.5 randomized to diet group (*n* = 39) and non-diet group (*n* = 39)	35.7 ± 3.6	Moderate CR and restriction of fat intake + LEARN program for weight control	HAES	Cognitive control of eating, loss of control susceptibility to perceptions of hunger (EI questionnaire). Drive for Thinness, Bulimia, and Body Dissatisfaction (EDI-2 questionnaire)	Both groups attended 24 weekly sessions, each 90 min in length. A 6-month aftercare program was available, described as optional monthly group support sessions	At the end of aftercare program. Attrition post-aftercare in the diet group was higher (41%), compared to non-diet group (8%)	There was weight loss only in the diet group and it was maintained at follow-up Cognitive restriction increased significantly in the diet group and decreased significantly in the non-diet group. For both groups, this change was sustained at follow-up. The variable disinhibition (loss of control that follows violation of self-imposed rules) improved significantly in the non-diet group compared with the diet group at follow-up
Bacon et al. [26]	USA	RCT	78 adult Caucasian women randomized to HAES group (*n* = 39, age 41.4 ± 3) and diet group (*n* = 39, age 40.0 ± 4.4)	HAES group: 35.9 ± 4.6 Diet group: 36.7 ± 4.2	Moderate CR and restriction of fat intake + LEARN program for weight control	HAES	Cognitive control of eating, loss of control susceptibility to perceptions of hunger (EI questionnaire). Drive for Thinness, Bulimia, and Body Dissatisfaction (EDI-2 questionnaire)	Both groups attended 24 weekly sessions, each 90 min in length. A 6-month aftercare program was available to each group, described as optional monthly group support sessions	52 weeks post-aftercare program. Almost half of the diet group dropped out (42%) before the end of treatment, whereas almost all (92%) of the HAES group completed the program	The HAES group members maintained weight and BMI throughout the study and follow-up period. The diet group significantly decreased their weight posttreatment but regained some of the weight such that weight was not significantly different between baseline and follow-up The diet group did not sustain the improved restraint at follow-up, while the HAES group members were able to sustain their decrease in restraint (and interoceptive awareness) at follow-up Both groups demonstrated significant improvement posttreatment on susceptibility to hunger and disinhibition (loss of control that follows violation of self-imposed rules). The HAES group maintained these improvements; the diet group maintained the improvement in disinhibition, but not hunger
Anglin et al. [24]	USA	RCT	16 adults both sexes aged 20–48, randomized to diet group (*n* = 8) and IE group (*n* = 8)	Diet group: 33.74 ± 2.08; IE group: 34.64 ± 2.32	CR of 500 kcal/day	IE	Not specified	Number and duration of sessions is not specified. The study lasted 6 weeks	None follow-up. Attrition rate note specified	The diet group had a significantly greater weight loss than the IE group The IE group initially lost more weight than the diet group. This was, however, reversed at the endpoint. Within the diet group, weight loss was steady and consistent and no statistically significant difference in the rate of weight loss between midpoint and endpoint was observed
Mesinger et al. [23, 29]	USA	RCT	80 women randomized to the WN program (*n* = 40, age 39.83 ± 4.34) or WL program (*n* = 40, age 39.35 ± 3.91)	WN program: 37.4 ± 0.61 WL program: 38.6 ± 0.61	CR + + LEARN program for weight control	HUGS program for better health	Restraint, Eating Concern, Weight Concern, and Shape Concern (EDE-Q)	Both groups attended 90-min weekly sessions for 6 months	18 months post-intervention. 26 participants in the WL program and 22 participants in the WN program were lost at follow-up	Between-group differences in weight loss and BMI change were observed from baseline to post-intervention (*p* = 0.001; *p* = 0.002, respectively), with greater reductions in the WL program post-intervention. However, these between-group differences in weight loss and BMI were no longer significant at 24 months (*p* = 0.063; *p* = 0.057, respectively) Within-group effects of time for global DEBs scores were evident only in the WN program. Participants reported reductions in global EDE-Q score at the 6-month assessment *(p* < 0.001), and these reductions were sustained at the 24-month assessment (*p* = 0.001)
Campos et al. [27]	Brazil	RCT	82 adults both sexes (mean age 50.5 ± 9.1) randomized to CG (*n* = 27), IEG (*n* = 28) and IEGDG (*n* = 27)	CG 47.5 ± 6.2 IEG: 47.6 ± 6.8 IEGDG: 50.2 ± 9.4	CG group: (20 kcal/kg/day, balance in macro and micronutrients) IEGDG group: Brazilian dietary guidelines	IE	Risk to develop binge eating disorder (BES); emotional eating, cognitive restriction, and uncontrolled eating (TFEQ-R21)	Number of sessions differed according to the intervention; the intervention lasted three months	3 months post-intervention. 4 participants in the CG program, 3 participants in the IEG program and 1 participant in the IEGDG program were lost at follow-up	There were no significant intragroup differences in the dimensions of eating behaviors, weight, or BMI
Pepe et al. [30]	Brazil	RCT	138 women randomized to: MER group (*n* = 49, age 36.6 ± 6.8); ME group (*n* = 46, age 37 ± 7.2) or ME + MER group (*n* = 43, age 36.4 ± 7.7)	MER: 32.5 ME: 34.4 ME + MER: 33.4	MER: deficit of 500 kcal/day ME + MER: deficit of 500 kcal/day	ME	Risk to develop binge eating disorder (BES); emotional eating, cognitive restriction, and uncontrolled eating (TFEQ-R21)	ME group and ME + MER group joined 7 sessions lasting 90 min once per month	6 months post-intervention. 18 participants (36.7%) in the MER program, 30 participants (65%) in the ME program and 23 participants (53%) in the ME + MER program were lost at follow-up	Weight loss was significant, but no statistically significant difference was found between the groups (*p* = 0.692) The association between ME with energy restriction did not promote greater weight loss than ME or MER. There was a greater reduction in uncontrolled eating in the ME group than in the MER group and a greater reduction in emotional eating in the ME group than in both the MER and the ME + MER groups. No statistically significant differences were found in the other variables evaluated between groups

*HAES* Health at every size; *IE* intuitive eating; *WN* weight neutral; *WL* weight loss; *HUGS* Health-focused, Understanding lifestyle, Group supported, and Self-esteem building; *CG* control group; *IEG* intuitive eating group; *IEGDG* Intuitive Eating and nutritional GuiDelines application Group; *MER* moderate energy restriction; *ME* mindful eating; *ME* + *MER* mindful eating + moderate energy restriction; *RCT* randomized controlled trial; *BES* Binge Eating Scale; *EI* Eating Inventory; *EDI-2* Eating Disorder Inventory—2; *EDE-Q* Eating Disorder Examination Questionnaire; *TFEQ-R21* Three Factor Eating Questionnaire reduced version with 21 items; *CR* calorie restriction

## Discussion

This narrative review has investigated the role of dietary versus non-dietary approaches on weight loss and DEBs in adults with obesity. Restrictive diets are the most commonly used approach for managing weight in subjects with obesity; however, while they are effective in the short term, numerous studies indicate that weight is often regained over time [8–11]. According to this aspect, a recent systematic review, examining the effects of nonsurgical and nonpharmacological interventions for maintaining long-term weight loss in adults with obesity, found that most high-quality follow-up treatment studies do not result in long-term weight loss [31]. Moreover, emerging research shows associations between restrictive diets and DEBs, while new evidence shows that non-dietary approaches such as ME, IE, and WN are more effective in promoting healthy eating behavior [32].

In the current narrative review, three non-dietary approaches are explored: ME, IE and WN programs, while all of the dietary-approaches are characterized by moderate CR; only one study [28] refers to a restriction of fat intake.

ME is considered only in the study by Pepe et al. [30] in which 138 women were randomized to the MER group, ME group and ME + MER group. All patients in the ME and ME + MER groups received audio recordings of the mindfulness exercises (e.g., breath mindfulness) via smartphone message or email for daily practice at home. In this study weight loss was observed in both the ME and ME + MER groups, but there was no statistical difference between the groups (Table 1). However, there was a greater reduction in uncontrolled eating in the ME group compared to the MER group and a greater reduction in emotional eating in the ME group compared to both the MER and the ME + MER groups.

Another study in which CR associated with mindfulness components is investigated is that of Daubenmier et al. [33]. This study was not included in Table 1 because it does not compare a dietary and a non-dietary approach, but rather compares a diet–exercise intervention with mindfulness components and a diet–exercise intervention without mindfulness components. The mindfulness components added included mindfulness training for stress management, eating, and exercise. These ME practices were designed to enhance awareness and self-regulation of physical hunger, stomach fullness, taste satisfaction, food cravings, emotions, and other eating triggers in the context of reduced caloric intake. This study reported greater weight loss, not statistically significant, at follow-up (12.5 months post-intervention) in the group following a dietary-approach associated with mindfulness components compared with the group following the standard dietary-approach.

The second non-diet approach that emerges from the included studies is IE, which was investigated in two of the included studies [24, 27]. Campos et al. [27] randomized 82 adults of both sexes to CG (receiving standard treatment for patients awaiting bariatric surgery), Intuitive Eating Group (IEG) and Intuitive Eating and nutritional Guidelines application Group (IEGDG). Both the IEG and IEGDG groups consisted of candidates for bariatric surgery, who were required to follow a strict dietary protocol as part of their post-operative procedure. The objective was to prevent the deprivation of certain foods from resulting in obsessive feelings of deprivation. Specifically, the IEG and IEGDG groups were taught the 10 principles of IE and were assigned home activities, such as watching videos reinforcing the principles “Reject the Diet Mentality”, keeping a food diary, practicing breathing exercises, completing checklists related to internal hunger and satiety cues, and engaging in ME during meals. At the end of the intervention, no significant differences in eating behavior and weight were found within the group during the intervention or at follow-up.

The second study that reported IE as a non-dietary intervention is that of Anglin et al. [24], in which the non-dietary approach focused on the ten principles of IE. Although the diet group experienced significantly greater weight loss than the IE group, it is unknown if this result was maintained long-term, as the study did not include a follow-up.

Finally, the third non-dietary approach that is reported in the review is the so-called WN approach; specifically, four studies [23, 25, 26, 28, 29] considered three different WN approaches: Bacon et al. [25, 26] investigated the Health at Every Size® (HAES®) program, Mensinger et al. [23, 29] reported on the HUGS program, while Goodrick et al. [28] reported on a WN program not specifically named.

Going into details, the HAES® approach advocates for transitioning from a weight-centric to a WN perspective, urging individuals of diverse body sizes to adopt healthier behaviors without primarily emphasizing weight loss. The values of HAES endorse the cultivation of enjoyable and sustainable physical activity routines, as well as adaptable, personalized eating patterns guided by hunger, satiety, nutritional requirements, and enjoyment [34]. This approach achieved positive outcomes on the eating behavior of subjects enrolled in both studies by Bacon and colleagues [25, 26]. In the first study [25] weight loss occurred only in the restrictive diet group and was maintained at follow-up, while disinhibited eating (loss of control following violation of self-imposed rules) improved significantly in the HAES group compared with the diet group at follow-up. In the subsequent study [26] participants of the HAES group maintained their weight and BMI throughout the study period and follow-up while the diet group significantly decreased weight after treatment, but regained some of the weight which was not significantly different between baseline and follow-up (Table 1). With regard to eating behaviors both the HAES and the diet groups showed significant improvement in hunger susceptibility and disinhibited eating after treatment, but the HAES group maintained these improvements, while the diet group maintained improvements in disinhibition but not in hunger [26].

The second WN approach that emerges from the included studies is the HUGS program for Better Health, which stands for Health-focused, understanding lifestyle, Group supported, and Self-esteem building [35]. HUGS is a holistic health promotion program that follows an evidence-based manualized curriculum incorporating the main components of HEAS and WN approaches. HUGS conveys principles emphasizing eating for well-being and pleasure and participating in physical activity for personal enjoyment and fulfillment. A primary objective of this program is to assist participants in breaking free from a dieting mentality, which frequently results in a harmful cycle of binge eating and feelings of guilt stemming from excessively restrictive lifestyles [35]. The study that compared this approach with a dietary- approach is that of Mensinger et al. [23] in which a greater weight reduction in the post-intervention phase was observed in the diet group. However, this difference was no longer significant at 24 months follow-up. Concerning eating behaviors, the women who participated in the WN program showed significantly greater improvements in DEBs from baseline to follow-up compared to the women who participated in the conventional WL program (Table 1).

Finally, Godrick et al. [28] also conducted a study in which a WN approach and a dietary approach were compared. The WN approach began with a psychotherapeutic phase to address psychological aspects of obesity, focusing on self-esteem and body image before dietary and exercise modifications. Instructors covered topics like the origins of restrictive dieting and overeating cycles, self-esteem issues, and the importance of supportive social networks. Subsequent sessions led by dietitians discussed weight management, the relationships between diet, mood, and behavior, and factors influencing overeating. In this study, the diet group lost significantly more weight than the non-diet group (*p* < 0.04), and both groups had similar reductions in binge-eating scores at 6 months. At follow-up, both groups had similar weight gains of over 1 kg above baseline and showed similar decreases in binge-eating scores from baseline.

In light of the findings from the comparative analysis of dietary and non-dietary approaches in weight management for individuals with obesity and DEBs, it is clear that weight loss in this population cannot be achieved without CR. However, the psychological improvements reported with non-dietary approaches compared to dietary ones should not be overlooked. This aspect is clinically significant, as healthcare providers must consider the possibility that patients with obesity may exhibit DEBs, which, if not adequately addressed, can complicate the management of the condition. Additionally, the social context in which the individual lives can exacerbate obesity, making the role of policymakers as important as that of clinicians. Raising public awareness through campaigns that emphasize the psychological aspects of obesity and DEBs is critical, as increased awareness can reduce the stigma associated with obesity by helping to create a supportive environment that can help combat this condition.

Given the complexity of obesity, its management requires a multidisciplinary approach that includes several strategies and programs involving synergistic actions between diet, exercise, and practices to improve DEBs.

## Conclusions

CR is essential for weight loss in individuals with obesity, but long-term weight management also hinges on their relationship with food. Many individuals with obesity exhibit DEBs that exacerbate their condition and hinder weight loss efforts. These challenges can be mitigated by adopting approaches that emphasize mealtime awareness, IE, and reducing internalized weight stigma. Future research should focus on developing multidisciplinary strategies that not only prioritize dietary restriction, but also consider the eating behaviors of individuals with obesity. This could include integrating psychological support, mindfulness-based interventions, behavioral interventions, nutritional education and IE to address the emotional and cognitive aspects of eating. Additionally, longitudinal studies are needed to evaluate the long-term effects of these interventions on both weight maintenance and psychological well-being. Investigating diverse populations across different cultural contexts will also enhance the understanding of how various factors influence eating behaviors and obesity management. By addressing these areas, future research can contribute to more effective, holistic strategies that promote sustainable weight loss and improve the quality of life for individuals facing obesity.

## Strength and limits

This narrative review has inherent limitations. The authors chose to include studies that compared a dietary approach with a non-dietary approach and to exclude studies comparing a non-dietary approach with a control group. This limited the number of studies that could be selected. Additionally, a single database was used, which may have resulted in the exclusion of some studies. The limited availability of such studies has resulted in the inclusion of a small number of studies, many of which have limited sample size. Additionally, an important gap that emerges from this review is the lack of classification of DEBs and the use of different validated tools for their investigation, in fact various instruments were employed, including the Binge Eating Scale (BES), Eating Inventory (EI), Eating Disorder Inventory-2 (EDI-2), Eating Disorder Examination Questionnaire (EDE-Q), and the Three Factor Eating Questionnaire reduced version with 21 items (TFEQ-R21). This variation in assessment tools may have contributed to discrepancies in the findings, as each instrument has its own unique focus and sensitivity to different aspects of eating behaviors. Consequently, the differing methodologies may impact the comparability of results and the overall conclusions drawn from the studies. Furthermore, there are few studies with follow-up periods longer than one year, making it challenging to accurately assess the long-term benefits of these different approaches. Finally, one notable limitation of this review is that all included studies were conducted exclusively in two countries, Brazil and the United States. This geographic concentration may limit the generalizability of the findings to other populations and settings. Future research should aim to include a more diverse range of countries and cultures to enhance the applicability of the results. A broader representation of participants will be crucial for understanding how these findings may vary across different demographic and cultural contexts. Nevertheless, our data add support to the existing literature on the use of CR in patients with obesity by emphasizing the need to develop interventions that aim to improve not only their eating habits, but also their relationship with food in order to achieve long-term weight loss.

## What is already known on this subject?

Obesity is a chronic condition characterized by excess body fat, significantly increasing the risk of numerous diseases and imposing an economic burden of approximately $2 trillion annually. Traditional restrictive diets, commonly used to combat this global epidemic, often result in weight regain and can trigger DEBs and EDs.

Emerging approaches such as ME, IE, and methods that promote a positive relationship with food and body image are showing promise. These approaches emphasize mindfulness during meals and respect for internal hunger and satiety signals. While they aim to improve overall eating behaviors and reduce BMI, further research is necessary to validate their effectiveness. This review conducts a comparative analysis of dietary and non-dietary methods for managing obesity and DEBs in adults.

## What this study adds?

The data obtained from this narrative review underscore that CR alone is not an effective long-term intervention for weight management in individuals with obesity. To achieve sustained weight loss, it is crucial to also address the DEBs often present in this population. This narrative review can provide insights for the development of multidisciplinary interventions for managing individuals with obesity and a disturbed relationship with food.

## Data Availability

No datasets were generated or analysed during the current study.
